# Prenatal Nicotine and Maternal Deprivation Stress De-Regulate the Development of CA1, CA3, and Dentate Gyrus Neurons in Hippocampus of Infant Rats

**DOI:** 10.1371/journal.pone.0065517

**Published:** 2013-06-13

**Authors:** Hong Wang, Marjorie C. Gondré-Lewis

**Affiliations:** Laboratory for Neurodevelopment, Department of Anatomy, Howard University College of Medicine, Washington, D.C., United States of America; Rosalind Franklin University, United States of America

## Abstract

Adverse experiences by the developing fetus and in early childhood are associated with profound effects on learning, emotional behavior, and cognition as a whole. In this study we investigated the effects of prenatal nicotine exposure (NIC), postnatal maternal deprivation (MD) or the combination of the two (NIC+MD) to determine if hippocampal neuron development is modulated by exposure to drugs of abuse and/or stress. Growth of rat offspring exposed to MD alone or NIC+MD was repressed until after weaning. In CA1 but not CA3 of postnatal day 14 (P14) pups, MD increased pyramidal neurons, however, in dentate gyrus (DG), decreased granule neurons. NIC had no effect on neuron number in CA1, CA3 or DG. Unexpectedly, NIC plus MD combined caused a synergistic increase in the number of CA1 or CA3 neurons. Neuron density in CA regions was unaffected by treatment, but in the DG, granule neurons had a looser packing density after NIC, MD or NIC+MD exposure. When septotemporal axes were analyzed, the synergism of stress and drug exposure in CA1 and CA3 was associated with rostral, whereas MD effects were predominantly associated with caudal neurons. TUNEL labeling suggests no active apoptosis at P14, and doublecortin positive neurons and mossy fibers were diminished in NIC+MD relative to controls. The laterality of the effect of nicotine and/or maternal deprivation in right versus left hippocampus was also analyzed and found to be insiginificant. We report for the first time that early life stressors such as postnatal MD and prenatal NIC exposure, when combined, may exhibit synergistic consequences for CA1 and CA3 pyramidal neuron development, and a potential antagonistic influence on developing DG neurons. These results suggest that early stressors may modulate neurogenesis, apoptosis, or maturation of glutamatergic neurons in the hippocampus in a region-specific manner during critical periods of neurodevelopment.

## Introduction

The experience of trauma, abuse, or neglect during infancy, puts individuals at risk for developing psychiatric diseases such as major depression, anxiety, hyperactivity, and posttraumatic stress disorder (PTSD) [1], [2], [3], [4], [5], and can also be a trigger for schizophrenia and borderline personality disorders later in life [2], [4], [5]. Experience of these early life stressors is also associated with increased risk for substance abuse, including heavy nicotine use [6], [7], a common behavior in patients who suffer from major depression, schizophrenia, and anxiety. In parallel, exposure to nicotine during gestation or early in life is a strong risk factor for developing anxiety, attention deficit hyperactivity disorder (ADHD), memory deficits and other cognitive and sensorimotor deficits [8], [9]. Early life stress in the form of maternal deprivation and prenatal drug exposure are prevalent in low-income households, and clinical research suggests that drug effects in children may be exacerbated when combined with multiple risk factors, including nonoptimal caregiving environments (reviewed in [10]).

During the pre- and peri-natal periods, the hippocampus undergoes extensive development; a process that involves active neurogenesis, neuronal maturation inclusive of synaptogenesis with target neurons, and synaptic stabilization [11], and is particularly susceptible to exogenous stressors [12]. Perturbation of neuronal development by exposure to drug or stressful situations is likely to disturb the expansion of neural progenitor cells, possibly by interfering with proliferation, migration and maturation of neurons, and may also affect the pruning of cell numbers via apoptosis.

In early studies of the hippocampus after gestationally restricted exposure to nicotine, the dendritic arbor, spine density and organelle content of CA1, CA3, and dentate gyrus (DG) were altered long-term in P40 rats [13]. More recently, we showed a substantial upregulation of subunits of NMDA and AMPA receptors and other proteins important for synaptic plasticity in newborn P1 rat pups [14]. In young adults, muted tissue levels of glutamatergic receptor subunits, accompanied with downregulated ^3^[H] AMPA binding of functional AMPARs in the hippocampus [14] is consistent with a reduction in LTP induction in gestational nicotine treated animals [15]. The reduction in LTP induction is attributable to reduced amplitude and frequency of AMPAR-mediated EPSCs after nicotine treatment [15], [16]. Such molecular and physiological changes in AMPAR support the impaired learning behavior observed when animals were challenged with performance of spatial tasks [16]. In fact, exposure to nicotine prenatally or during the early postnatal period is linked to a higher level of anxiety-like behavior in adults [17] and altered exploration, novelty seeking behavior, and blocked extinction learning in fear conditioning behavioral paradigms [15], [16], [17]. Depressive-like behaviors are preponderant in prenatal nicotine exposed rat pups as early as 3-weeks of age [15] and later in full-fledged adults [15], [16]. These studies support the idea that prenatal nicotine modulates hippocampus-linked emotional as well as intellectual behavior in young and older offspring.

Maternal separation during early life delays the developmental switch from NR2B-containing NMDA receptors to NR2A NMDARs [18], [19], and renders hippocampal neurons more susceptible to long-term depression of fEPSPs during infancy and adolescence. By contrast, in adults who previously experienced maternal deprivation (MD), there is a downregulation of NR2A, NR2B, and AMPA receptor subunits GluR1 and GluR2 [20], [21]. BDNF levels can be modulated upward or downward by MD depending on the MD paradigm used and the age at which levels were examined [22], [23], [24]. Although the mechanisms are not yet elucidated, there appears to be an intimate relationship between stress and many molecules associated with learning and memory [24], [25], [26], [27].

Modification of synaptic molecules and neurotransmission may be governed by structural alterations in neuron number and early processes involving neurogenesis or apoptosis. Although MD has been shown by multiple groups to decrease neurons in the adult DG [28], [29], pre-natal nicotine exposure did not significantly affect neuron number or volume of adult CA1, CA3 or DG [30].

We introduce a new model to investigate the combined effect of drug exposure (prenatal nicotine (NIC)) and stressful experiences (postnatal maternal deprivation (MD)) during development and long-term in the adult. In humans, these conditions are likely experienced together in the infant, and nothing is known about the short and long term effects of the combination of the two on the brain or how early in postnatal development the effects can be detected.

Our findings show that developing CA1 was sensitive to developmental perturbations, and this sensitivity resulted in increased pyramidal neuron numbers at P14, whereas developing DG was primarily sensitive to the MD treatment, which resulted in decreased neuron numbers. The novel findings reported in this study provide evidence of a greater than additive effect of maternal deprivation combined with prenatal nicotine exposure in CA1 and CA3 at P14, and these synergistic effects can be specifically attributed to the rostral hippocampus. MD+NIC exposure alters the neuronal contribution of individual subregions to total hippocampal neurons, and this may be due to changes in neurogenesis.

## Materials and Methods

### Animals and Prenatal Nicotine Treatment

This study was carried out in strict accordance with the recommendations in the Guide for the Care and Use of Laboratory Animals. The protocol for this study (MED-10-05) was approved by the Institutional Animal Care and Use Committee of Howard University. Surgical procedures were performed under isoflurane anesthesia, and all efforts were made to minimize suffering. Prenatal nicotine treatment was performed as previously published [14]. Timed-pregnant Sprague-Dawley rats weighing 250–300 g (Harlan Laboratories, Frederick, MD, USA) were housed under 12 h light/dark cycles, with free access to food and water. Nicotine was prepared fresh on the day of pump implantation in 0.9% saline, and the pH was adjusted to 7.4–7.6. This dose is commonly used to achieve circulating blood nicotine levels of a 2–4 packs/day smoker [31]. Pumps hold a volume of ∼250 µl and have a flow rate of ∼0.5 µl/h. On gestational day 7 (G7), pregnant dams were surgically implanted between the scapulae with a mini-osmotic infusion pump (model #2004) (Alzet, Cupertino, CA, USA), containing either 0.9% saline or nicotine hydrogen tartrate (Sigma- Aldrich, St. Louis, MO, USA) which was delivered at a rate of 4 mg/kg/day until parturition at G21/P0 [14], [32]. Dam weights were monitored daily, and on G21, pumps were removed by aseptic surgery. To examine the effects of a combined pre- and post-natal exposure to nicotine on pup weight and development, an additional group (PP-NIC) was analyzed. PP-NIC pups were exposed to nursing mothers with nicotine pumps implanted until weaning day, i.e., postnatal day 21 (P21) (Alzet mini-osmotic pump Model #2006).

### Maternal Deprivation Regimen

The maternal deprivation paradigm was developed after analysis of other published protocols [20], [21], [28], [33]. Beginning at P2 until weaning at P21, pups were removed from their mothers’ home cage, moved to a different room altogether and exposed to maternal deprivation daily for 3 hours from 11 am to 2 pm. The MD room temperature was monitored and maintained at 29°C with a heater to simulate the warmth of the mother’s body. After the 3 hours, the pups were returned to their mothers.

For experimental comparisons, whole litters were assigned to one of four groups: controls (CTL) received Alzet pumps filled with saline and were kept with their mothers during the entire postnatal period as customary; maternally deprived groups (MD) received saline during the prenatal period and at P2, underwent the maternal deprivation procedure until P14 or P21; nicotine groups (NIC) received nicotine prenatally as described above, and were kept with their mothers from birth until sacrificed; maternal deprivation plus nicotine groups (NIC+MD) were first exposed to prenatal nicotine followed by the maternal deprivation paradigm postnatally. Within each treatment group, numbers (*n*) used for stereology represent 5 animals (3 males and 2 females) from 3 different litters per group. Animals used for weight analysis are described further under “Weight Inspection”. Another category of animals only used for weight analysis received continual exposure to nicotine during the pre- and into the post-natal period (PP-NIC) until weaning.

### Weight Inspection

Weight determination studies include approximately 70 different animals from at least 4 dams per group. Pups were weighed every other day starting at P2 until P21 and every 4 days thereafter until P68. Males, avg n = 19 per group at P2–P4, n = 15 per group at P6–P14, n = 10 per group at P16–21, n = 6 per group from P24–P68. Females, avg n = 20 per group at P2–P4, n = 15 per group at P6–P14, n = 10 per group at P16–P20, n = 6 per group from P24–P68. PP-NIC averaged 5–9 per group from P1–P14, and 3–5 per group from P16 to P24.

### Perfusion and Histology

Histological procedures and analysis were conducted on P14 pups. Animals were perfused according to Gondré-Lewis et al., with modifications [34]. Pups were deeply anesthetized with Isothesia (Isoflurane) (Butler Animal Health Supply, Dublin, OH) and when insensate, underwent a thoracotomy for transcardial perfusion with 0.9% NaCl to clear the blood in the circulatory system. This was followed with a slow perfusion with chilled 4% paraformaldehyde (PFA) in 0.1 M phosphate/4% sucrose buffer, pH 7.4. Brains were post-fixed overnight in the 4%PFA/4% sucrose/0.1 M phosphate buffer, and then transferred to and stored in phosphate buffered saline (PBS), pH 7.4, at 4°C until ready for processing. Brains were freeze-sectioned on a sliding microtome to generate free-floating 50 µm coronal sections, and every 9^th^ section was mounted onto gelatin-coated slides, and dried overnight for staining with cresyl violet for the stereology studies. A different set of brains was used to generate 30 µm free-floating sections for immunocytochemistry studies and 10 µm slide-mounted sections for the TUNEL assays. Four to six sections of the hippocampal region from each of 2 CTLs or 3 MD+NIC groups were evaluated. The MD+NIC experimental group was used to analyse neurogenesis and apoptosis because if these events were ongoing, they would be readily detected.

### Doublecortin Immunocytochemistry

To detect immunoreactivity with the peroxidase method, endogenous peroxides were quenched with 0.3% H_2_O_2_ for 30 minutes. Sections were washed in PBS, permeabilized with 0.3% TX-100, blocked with 3% NGS/1%BSA for 1 hr, incubated with 1∶500 rabbit anti-DCX overnight (Abcam, Inc., Cambridge, MA), washed, and incubated with biotinylated goat anti-rabbit IgG. This was followed by exposure to ABC Elite reagent for 1 hr and subsequent incubation with DAB (Vector Laboratories, Burlingame, CA). Sections were mounted on gelatin-coated slides, and serially dehydrated with increasing concentrations of alcohol (70%, 90%, 95%, 100%) followed by Histosol. Slides were coverslipped in DPX mounting reagent. Images were taken with a Zeiss Axio Oberver Z1 (Zeiss, Gottingen, Germany) equipped for phase contrast light microscopy.

### TUNEL Staining

The ApopTag fluorescein in situ apoptosis detection kit (Millipore Corp, Cat # S7110) for terminal deoxynucleotidyl transferase dUTP nick end labeling (TUNEL) was used to assay for DNA fragmentation. Briefly, slide-mounted sections were washed and permeabilized in 0.3% Triton-X 100, incubated with digoxigenin (Dg) labeled nucleotides (Dg-DNTP) in the presence of terminal deoxynucleotidyl transferase (TdT) for 1 h at 37°C. Subsequently, the reaction was stopped, sections were washed and subjected to fluorescein conjugated anti-Dg antibody for 30 min. Positive controls were made by treating sections with 5000u/mL DNase I (Worthington Biochemical Corp., Cat #LS002139) for 10 min at RT prior to exposure to Dg-dNTP. Sections were counterstained with 0.2 µg/mL Hoescht 33258 (Invitrogen, Cat #H3570) and coverslipped in anti-fade reagent Fluorogel (Electron Microscopy Sciences, Cat#117985). Images were taken with a Zeiss Axio Oberver Z1 epifluorescence system equipped with an AxioCam MrM camera and AxioVision 4.8 Software (Zeiss, Gottingen, Germany).

### Stereological Analysis

**Figure 1:**
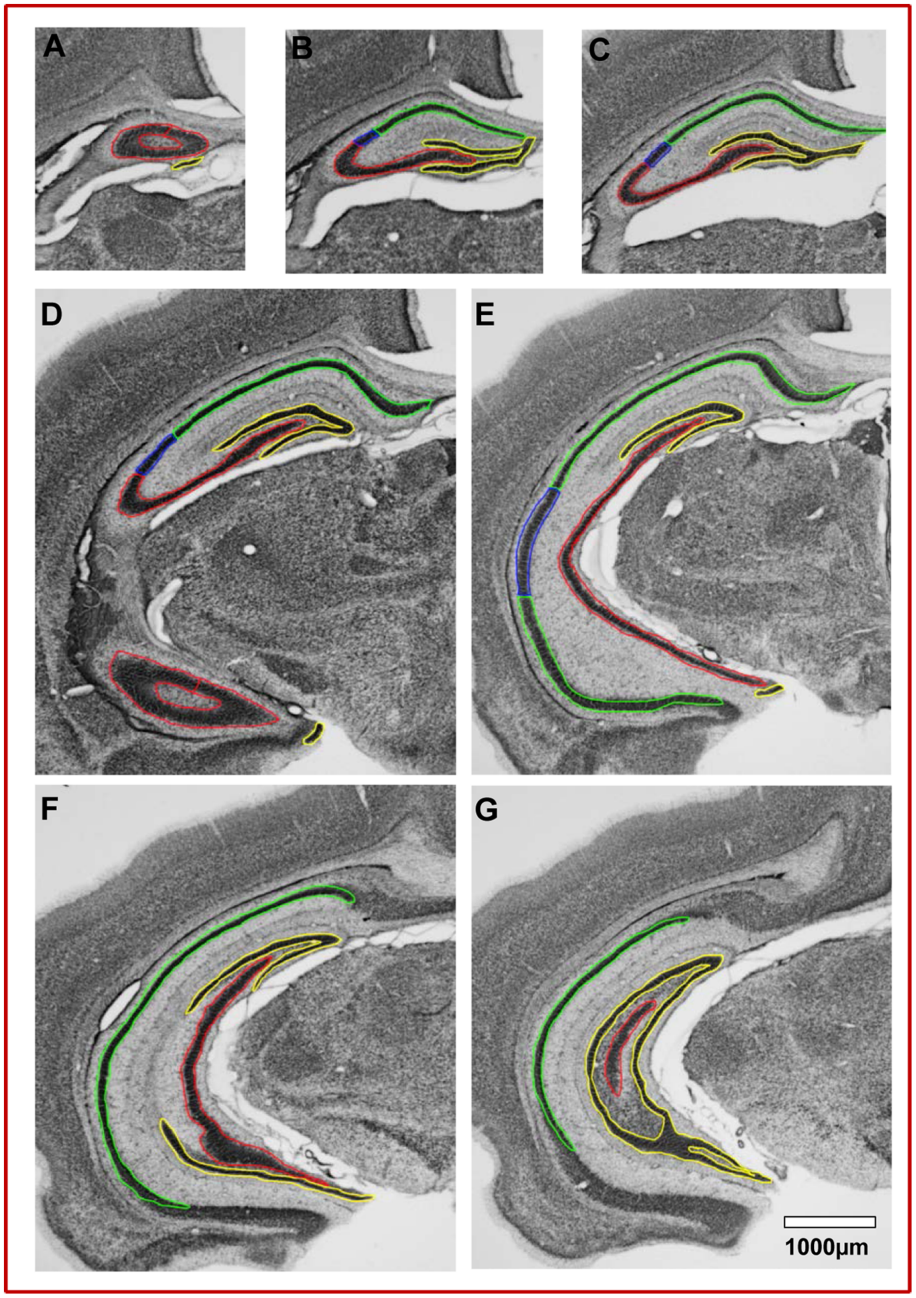
Rostral to caudal morphology of the rat hippocampus at P14. Representative example of the 1X sections used from a single rat brain. Coronal images are stained with Nissl and have a periodicity of 400 µm from the rostral-most (Fig. 1A) to the caudal-most section (Fig. 1F) of the hippocampus. The sub-regions, CA1, CA2, CA3, and DG, used for the stereology study are outlined (*green, CA1; blue, CA2; red, CA3; yellow, DG*). Regional boundaries were determined by cross-referencing with the atlases of Paxinos and Watson (2005) and Ramachandra et al. (2011). Bar = 1000 µm.

Unbiased stereology [35], [36], [37] was used to estimate mean total number, volume and density of pyramidal neurons in CA1, CA2, and CA3 subregions and granule neurons in the DG. All data collection was carried out using the StereoInvestigator software (MicroBrightfield, Inc., Colchester, VT). The system hardware consisted of an X–Y–Z motorized stage, an Optronics color video camera interfaced to a Nikon E800 microscope, a high-resolution video card, and a focus measurement encoder, which provides 0.25 µm resolution of absolute microscope stage focus position. The optical fractionator method systematically samples the region of interest to estimate total population numbers too large to count comprehensively [35 [37]. To use this method, the cell body layers of CA1, CA2, CA3 and DG subregions – over the entire rostral to caudal length of both rat hippocampi of each animal– were outlined using a 4X objective (Fig. 1). The CA cell body layer is enriched for pyramidal neurons whereas the DG cell body layer is enriched for granule neurons. The borders of CA1, CA2, CA3, and DG hippocampal subregions containing neuronal cell bodies were defined according to an atlas of the neonatal rat brain [38] and the stereotaxic rat brain atlas [39]. The length of the P14 hippocampus at an interval of 400 µm yielded an average of 8 sections per brain for use in the stereological study.

Neurons were counted using high power oil-immersion objectives; 60X for CA subregions and 100X for DG, 1.4 numerical aperture. Sampling grid sizes (200 µm×200 µm for CA1 and CA3, 75 µm×75 µm for CA2, and 150 µm×150 µm for DG) and counting frames (30 µm×30 µm for CA regions and 15 µm×15 µm for DG) were optimized to achieve a mean Gundersen coefficient of error (CE) of <0.1 (CE range = 0.01 to 0.05), using systematic and random sampling. A guard volume 2.0 µm deep on both sides of the section was used to avoid introduction of errors due to sectioning artifacts, including uneven section surfaces and lost caps. Sampling grid sizes and counting frames were determined such that a minimum of 400 cells was counted in each brain area analyzed. In most cases, the number of cells counted per brain was significantly higher. The appropriate grid size and counting frames were determined based on results of pilot studies for each region. Neurons within the pyramidal or granule cell enriched layers were identified as Nissl positive cell bodies containing a nucleolus clearly in focus within the counting frame, with lightly stained surrounding cytoplasm. Neurons were further distinguished by size and their morphology. Therefore, although rare, smaller glial-shaped cells were not counted. The reference volumes for CA and DG regions were estimated using the Cavalieri principle with point counting. The density was calculated based on the mean total population numbers and reference volumes.

For analyses of rostral versus caudal hippocampus, for a given P14 animal, sections with dorsal hippocampus only, equivalent to sections of adult hippocampus at or anterior to Bregma −4.2 [39] were included in the rostral/dorsal group. Sections with any ventral subregions visible, equivalent to sections of adult hippocampus at or posterior to Bregma −4.36 [39] were included as part of caudal/ventral group. This yielded a fairly even number of rostral and caudal sections for comparison in each animal.

### Statistical Analysis

A one-way ANOVA was used to analyze and obtain statistics of neuron number, reference volume, or cell density at CA or DG subregions. Where the F-test showed a difference, a Newman-Keuls multiple comparison *post-hoc* test was applied for further comparisons. A two-way ANOVA followed by the Bonferroni *post-hoc* test was used to analyze animal weights at each time point (summarized in Table S1). A two-way ANOVA followed by the Bonferroni *post-hoc* test was used to analyze laterality (left/right) or regional (rostral/caudal) preponderance of treatments. In each case, the side or the region of the hippocampus and the treatment paradigm were independent variables. The number, volume or density were dependent variables, and were analyzed separately for CA1, CA2, CA3 or DG. The different subregions of the hippocampus could not be included as independent variables for comparison because of the large differences in cell numbers within these subregions. The descriptive statistics are displayed as mean ± standard error of the mean (SEM).

## Results

### Effect of Nicotine and Maternal Deprivation on Body Weight

**Figure 2:**
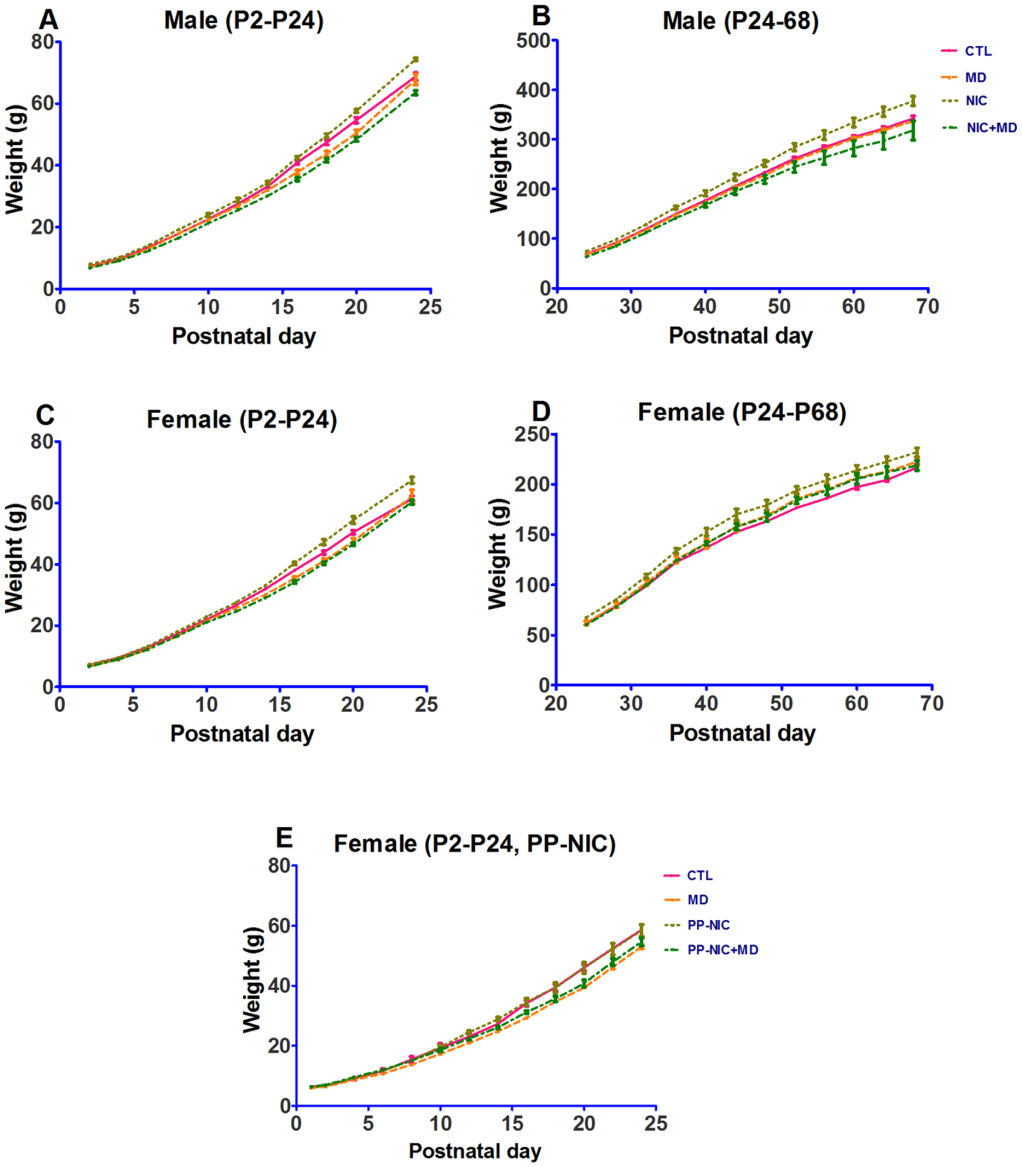
Comparative growth of nicotine-exposed and/or maternally deprived rat pups from P2–P70. The weight trend of male (A, B) and female (C, D) pups from P2–P24 (A, C) and P24–P70 (B, D) is shown. In E, animals were exposed to prenatal and postnatal nicotine (PP-NIC) until P21 when they were weaned. MD and MD+NIC significantly reduced animal weight, in the P2–P24 groups compared to CTLs. Please see tables 1 and S1 for significance values.

During postnatal development and prior to weaning, the body weight for male (Fig. 2A) or female (Fig. 2C) animals exposed to MD (MD only or NIC+MD) significantly decreased at P14, P16, P18, P20 compared to saline-exposed, non-maternally deprived animals (CTL or NIC), regardless of sex (see table 1 and table S1). NIC animals generally thrived and weighed more than CTLs, a difference that was maintained through adulthood (Fig. 2). After weaning, both NIC+MD and MD animals grew at a faster rate (rate data not shown) than either CTL or NIC animals such that there was no statistical difference compared to the untreated control in adulthood for either sex (Fig. 2B, 2D). These data show that early postnatal growth is depressed as a result of MD treatment, and accelerated growth rate after weaning compensatorily redirects MD and NIC+MD pup weights toward control levels, albeit more robustly in females (Fig. 2B vs. 2D). Next, we investigated if animals responded differently to prenatally restricted versus continuous exposure to nicotine during the pre- and post-natal period (PP-NIC, Fig. 2E). PP-NIC pups grew similarly to controls, but NIC+MD animals treated with the PP-NIC paradigm (Fig. 2E) still exhibited a depressed weight mirroring the MD alone, and significantly different from CTL or PP-NIC alone. Overall results of the ANOVAs are shown in table 1, and for each time point in table S1.

**Table 1:** Significance table for weight: whole group comparisons of developing rats.

	Male (P2–P24)	Female (P2–P24)	Male (P24–P68)	Female (P24–P68)	Female (PP-NIC, P2–P24)
MD vs CTL	*	*	ns	***	***
CTL vs NIC	*	**	***	***	ns
NIC+MD vs CTL	***	**	***	***	**
MD vs NIC	***	***	***	***	***
NIC+MD vs MD	*	ns	***	ns	*
NIC+MD vs NIC	***	***	***	***	**

CTL, control; MD, maternal deprivation; NIC, prenatal nicotine only; MD+NIC prenatal nicotine and maternal deprivation; PP-NIC, the NIC treatment consists of prenatal nicotine+postnatal nicotine until P21. Asterisks represent a significant reduction in weight of the first group.

ns, p>0.05;

* , p<0.05;

** , p<0.01;

*** , p<0.001.

### The (a)symmetry of Neuron Numbers in the Hippocampus

**Figure 3:**
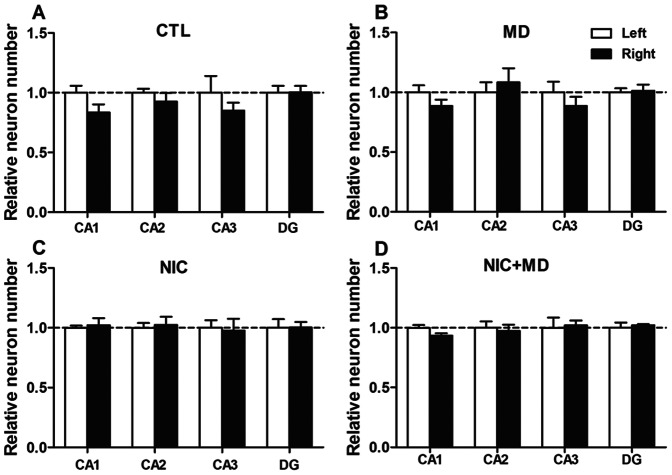
Comparison of CA1, CA3 and DG neurons in left or right hippocampus to determine regional laterality in P14 brains. Neuron numbers were evaluated from left (white) or right (black) hippocampi in CTL (A), MD (B), NIC (C), or NIC+MD (D). CTL and MD show a non-significant decrease in right CA1 and CA3 (A, B), but not for NIC or NIC+MD (C, D).

After spatial memory tasks, it is reported that gene expression predominantly changes in the right dorsal hippocampus and not the left, indicating a laterality in some hippocampus-dependent function [40], [41]. To test whether there was laterality to how left or right hippocampal neurons respond to prenatal nicotine or postnatal stress treatment paradigms, we analyzed pyramidal neurons from CA1 and CA3, and granule neurons from the DG. In control animals, the right CA1 and CA3 consistently had fewer pyramidal neurons than the left by −16.6±6.8% and −14.9±6.5% respectively, but these were not statistically significant (Fig. 3A). In MD animals, neurons were also non-significantly reduced in right CA1 by −11.4±5.2%, and in CA3 by −11.5±7.6% (Fig. 3B). Prenatal nicotine treatment had no effect on laterality in any hippocampal region (Fig. 3C). Neurons in right CA3 were no different from the left after NIC+MD exposure (Fig. 3D), and likewise, there was no difference in neuron number of left or right DG for control or any treatment group (Fig. 3, right bars). Measured reference volumes for left or right hippocampus was not significantly different, although a trend toward a lesser volume for right CA1 and CA3 but not DG was detected (data not shown). In summary, these data show no statistical difference between the left and right hippocampus of controls, NIC, or MD groups. Nonetheless, there was a trend toward fewer neurons on the right than the left in CA1 and CA3 of controls. These findings support the choice to use a single side consistently for stereological analysis. The data reported in the remainder of this study will represent total numbers and volumes encompassing both left and right hippocampi.

### Effect of Prenatal Nicotine and Maternal Deprivation on the CA1 Region of Hippocampus

**Figure 4:**
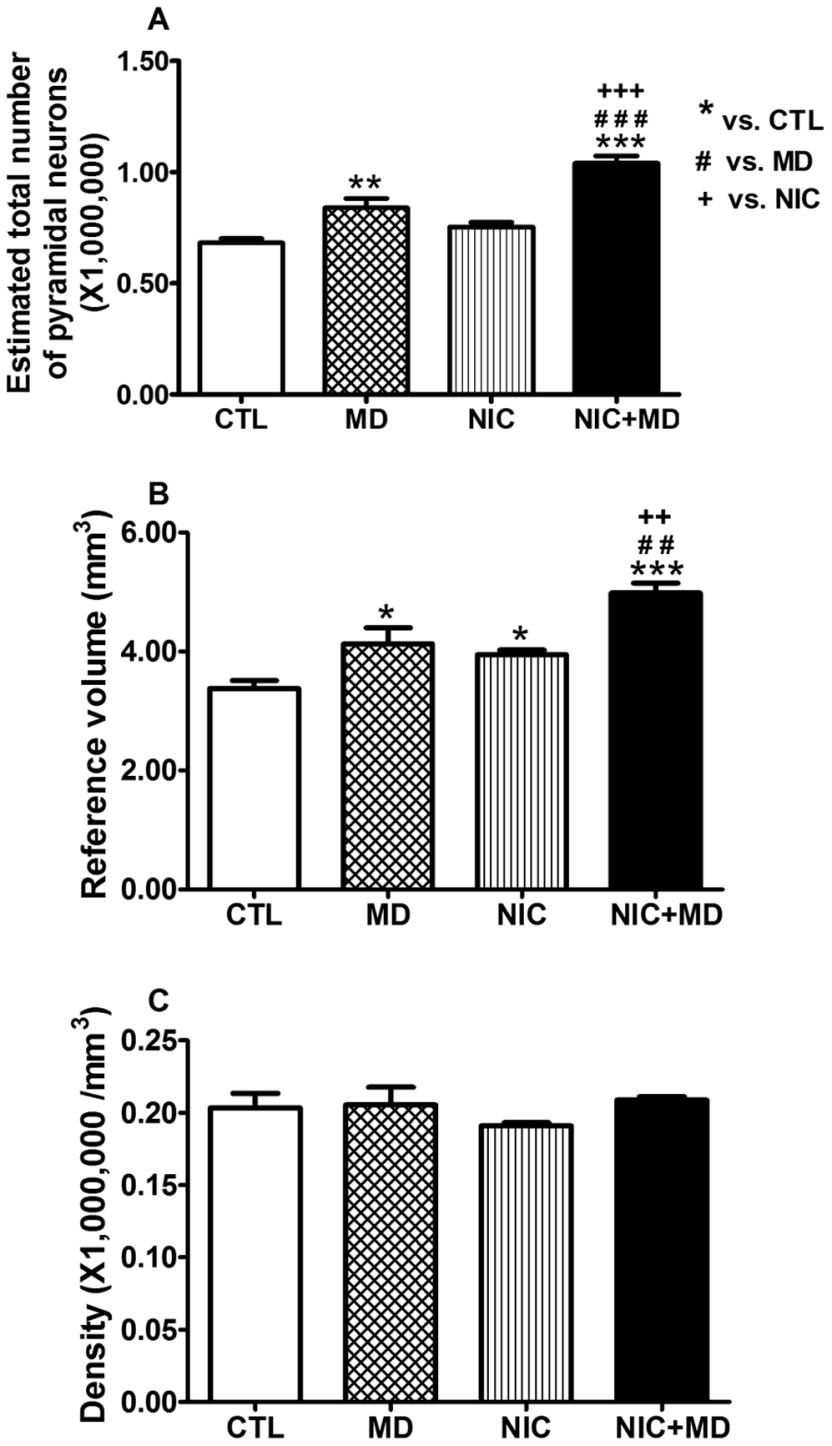
Analysis of pyramidal neuron number, volume, and density in CA1 of P14 rat hippocampus after prenatal nicotine exposure and/or maternal separation. After treatment with MD, NIC, and NIC+MD, CA1 pyramidal neuron numbers were elevated (MD, 23.1±6.2%, *p<0.01*; NIC, 10.4±2.9%, *p>0.05*; NIC+MD, 52.4±4.7%, *p<0.001*). Neurons of the NIC+MD group were significantly increased compared to MD or NIC alone (*p<0.001* for both) (A). The reference volume for MD, NIC and NIC+MD groups were significantly increased in comparison to CTL (MD, 22.2±8.0%, *p<0.05*; NIC, 16.9±2.4%, *p<0.05*; NIC+MD, 47.6±4.7%, *p<0.001*). The reference volume for the NIC+MD treated group was elevated compared to MD or NIC (*p<0.01 for both*) (B). Neuronal densities were not affected (p>0.05, C). *p<0.05; **/##/++ p<0.01; ***/###/+++ p<0.001.

Although CA1 and CA3 function as part of the whole hippocampus to acquire, process, and retain new memories, much research is focused on identifying distinct roles for each CA region in these processes. Thus, we traced the boundaries for CA1, CA2, CA3, and the dentate gyrus as in Fig. 1 from the rostral-dorsal most regions to the caudal-ventral most regions of the hippocampus to determine the treatment effect of NIC and/or MD on each subregion of the hippocampus. Normal developing P14 rats had 0.68×10^6^±0.02×10^6^ neurons in CA1, 0.41×10^6^±0.02×10^6^ in CA3, and 2.03×10^6^±0.11×10^6^ in DG bilaterally (see table 2). These data are fairly consistent with published reports of hippocampal neuron numbers for Sprague Dawley rats [42], [43], although our CA1 and CA3 numbers at P14 rats are slightly higher than those reported for adolescent P30 or adults, possibly due to incomplete pruning of neurons in those regions at P14. NIC did not significantly alter CA1 neuron number whereas MD increased the number of pyramidal neurons in this region by 23.1±6.2%, p≤0.01. When subjected to both NIC and MD stress, there was a 52.4±4.7%, p<0.001, increase in the number of pyramidal neurons in CA1 compared to CTLs (Fig. 4A). This number was greater than the sum of neuron alterations in either NIC or MD alone. Therefore, this finding suggests that when combined, exposure to prenatal nicotine and postnatal stress had a synergistic effect on CA1 neuron number in the developing hippocampus.

**Table 2:** Estimated pyramidal or granule cell numbers, volume and density for CA1, CA3 and DG of Sprague Dawley rat hippocampi at P14 (bilateral) after maternal deprivation and/or prenatal nicotine exposure.

•Estimated total number of pyramidal/granule cells (x 1,000,000)												
Brain region	CTL			MD			NIC			NIC+MD		
CA1	0.68±0.02			0.84±0.04**			0.75±0.02			1.04±0.03***, ###, +++		
CA2	0.07±0.001			0.06±0.01			0.07±0.003			0.07±0.003		
CA3	0.41±0.02			0.46±0.02			0.44±0.03			0.58±0.03***, ##, ++		
DG	2.03±0.11			1.74±0.03*			1.85±0.09			1.71±0.04*		
CA1-rostral	0.16±0.01			0.17±0.02			0.19±0.03			0.29±0.04*, #		
CA1-caudal	0.53±0.02			0.67±0.05*			0.56±0.04			0.73±0.05***, ++		
CA3-rostral	0.22±0.03			0.26±0.03			0.22±0.02			0.35±0.04*, +		
CA3-caudal	0.19±0.04			0.20±0.03			0.21±0.03			0.23±0.04		
DG-rostral	0.66±0.06			0.57±0.01			0.65±0.03			0.59±0.05		
DG-caudal	1.37±0.09			1.17±0.03*			1.21±0.10			1.12±0.05**		
**•Reference volume (mm^3^)**												
**Brain region**		**CTL**			**MD**			**NIC**			**NIC+MD**	
CA1		3.38±0.14			4.13±0.27*			3.95±0.08			4.98±0.16***, ##, ++	
CA2		0.28±0.01			0.27±0.02			0.28±0.01			0.30±0.02	
CA3		2.57±0.13			2.71±0.15			2.64±0.14			3.24±0.12*, #, +	
DG		2.27±0.12			2.34±0.10			2.36±0.06			2.45±0.07	
CA1-rostral		0.76±0.06			0.82±0.07			0.97±0.14			1.27±0.17	
CA1-caudal		2.62±0.12			3.31±0.23**			2.98±0.16			3.59±0.25***, ++	
CA3-rostral		1.41±0.18			1.54±0.09			1.49±0.13			1.89±0.18	
CA3-caudal		1.16±0.19			1.18±0.20			1.23±0.14			1.35±0.22	
DG-rostral		0.75±0.05			0.73±0.05			0.85±0.04			0.83±0.07	
DG-caudal		1.52±0.11			1.61±0.07			1.51±0.08			1.61±0.07	
**•Neuronal density (X 1,000,000/mm^3^)**												
**Brain region**			**CTL**			**MD**			**NIC**			**NIC+MD**
CA1			0.20±0.01			0.21±0.01			0.19±0.002			0.21±0.002
CA2			0.24±0.004			0.24±0.01			0.26±0.003			0.23±0.01
CA3			0.16±0.01			0.17±0.01			0.17±0.01			0.18±0.01
DG			0.90±0.05			0.75±0.04*			0.78±0.03*			0.70±0.02*
CA1-rostral			0.21±0.01			0.20±0.01			0.20±0.01			0.23±0.01
CA1-caudal			0.20±0.01			0.21±0.02			0.19±0.01			0.20±0.004
CA3-rostral			0.16±0.01			0.16±0.01			0.15±0.01			0.18±0.01
CA3-caudal			0.16±0.02			0.17±0.004			0.17±0.01			0.17±0.01
DG-rostral			0.89±0.06			0.80±0.06			0.77±0.03			0.71±0.02*
DG-caudal			0.91±0.04			0.73±0.04*			0.79±0.03			0.70±0.03**

* Values represent mean ± standard error of the mean (sem); (*) indicates significance compared to control, (#) compared to MD, (+) compared to NIC; */*#/+, p≤0.05;*

** */##/++, p≤0.01;*

*** */###/+++, p≤0.001.*

To test whether this increase in neurons affected the packing density, and possibly the neuroarchitecture of the hippocampus, we measured the volume occupied by CA1 neuronal perikarya. The CA1 volume of NIC, MD, and NIC+MD-exposed P14 pups increased to a similar extent as the cell numbers by 16.9±2.3%, 22.2±8.1%, and 47.6±4.9% percent, respectively (Fig. 2B); therefore yielding a comparable neuronal density to controls, at approximately 200,000 cells/mm^3^ (Fig. 4C, Table 2).

### Effect of Prenatal Nicotine and Maternal Deprivation on the CA3 Region of Hippocampus

**Figure 5:**
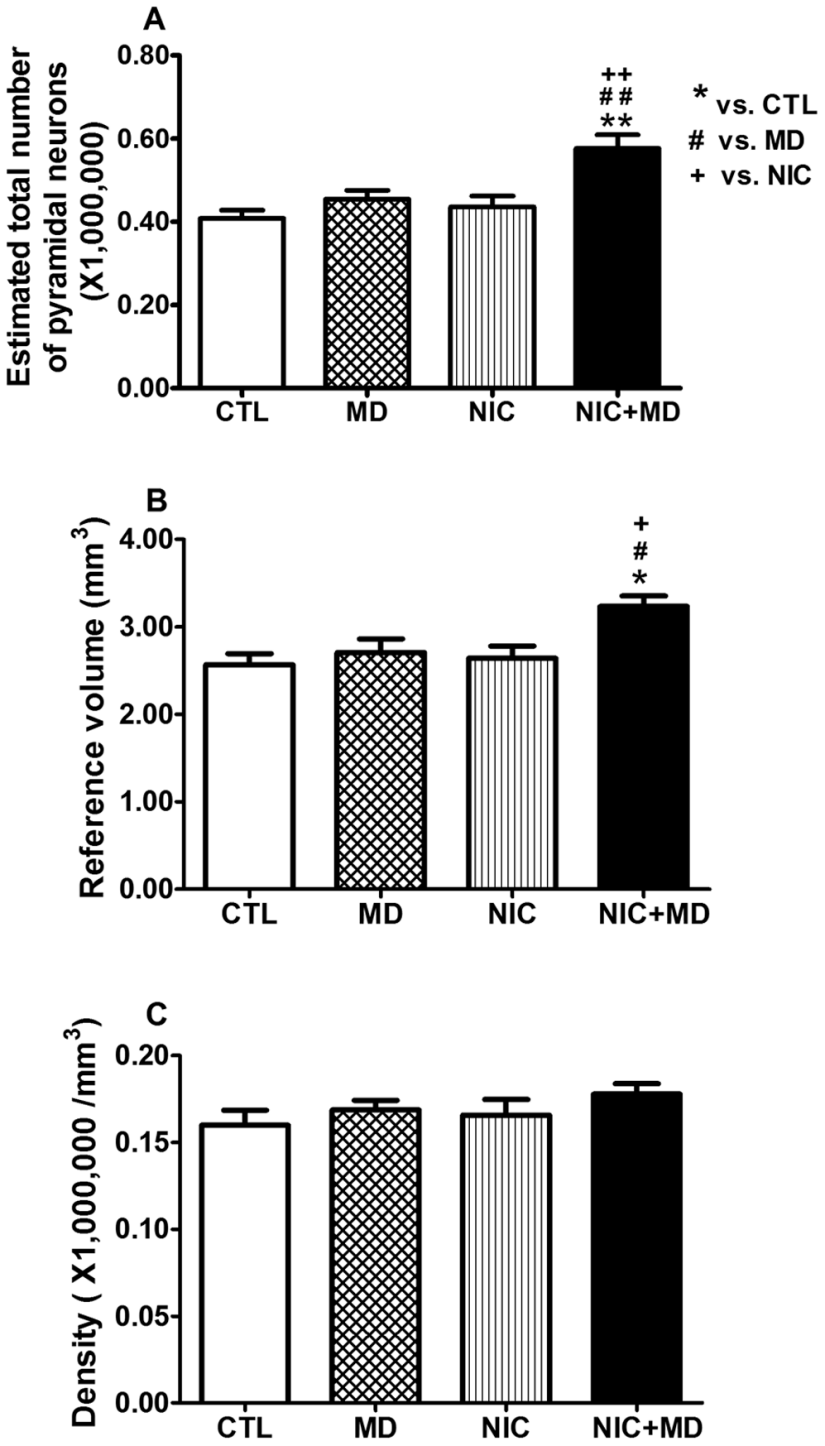
CA3 pyramidal neuron number, volume, and density estimations in P14 rat hippocampus following prenatal nicotine exposure and/or maternal separation. MD or NIC alone did not alter neuron number (A) and volume (B), however when combine NIC+MD neurons were significantly increased by 41.3±8.0%, *p<0.01* (A), and the reference volume was increased by 26.2±4.7%, *p<0.05*(B). Neuronal density was not different between groups (C). */#/+ p<0.05; **/##/++ p<0.01.

Given that CA3 fibers project onto CA1 neurons, we assessed changes in CA3 neuron number to determine whether MD and/or nicotine had the same effect on neuron development in CA3 as they did in CA1. Unlike CA1, CA3 neuron numbers were not affected by NIC or MD treatment alone, 6.9% and 12.8% increase, respectively, p>0.05 for both, but when NIC was combined with MD, CA3 pyramidal neurons increased by 41.3±8.0%, p<0.01, greater than the sum of the individual treatments (Fig. 5A). Like in CA1, the volume measured in this area followed a trend similar to the estimated neuron numbers, i.e., not significant for NIC or MD, but NIC+MD combined showed a 26.0±4.6% increase in volume, p<0.05 (Fig. 5B). There was no net change in neuronal density for any treatment paradigm (Fig. 5C, table 2).

### Prenatal Nicotine and Maternal Deprivation Reduce Granule Neurons in the Dentate Gyrus

**Figure 6:**
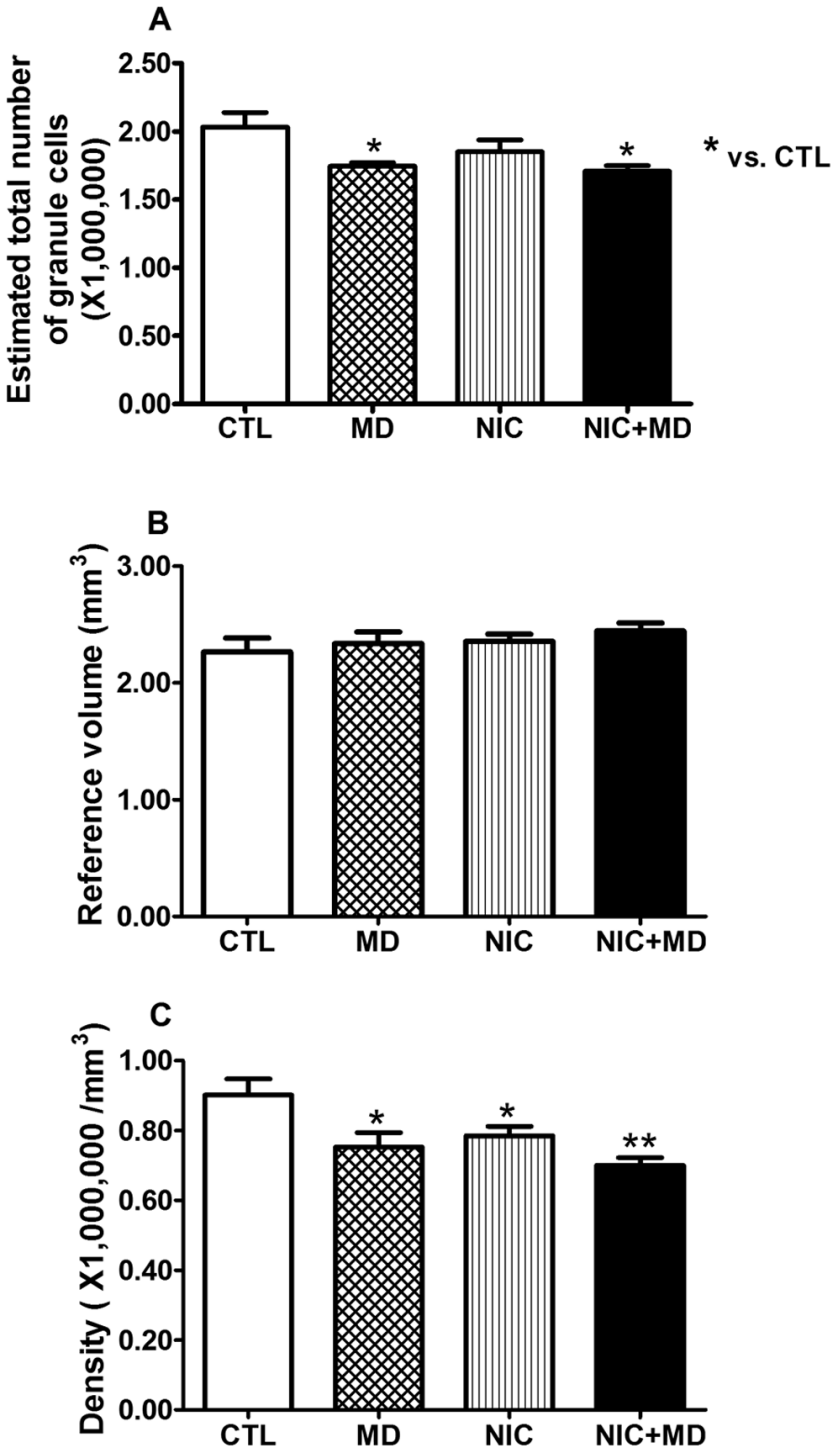
DG granule neuron number, volume, and density estimations in P14 rat hippocampus upon prenatal nicotine exposure and postnatal maternal separation. Compared to CTL, granule cell numbers of MD and NIC+MD but not NIC groups were significantly decreased (MD, 14.1±1.4%, *p<0.05*; NIC+MD, 15.9±2.0%, *p<0.05*) (A). Reference volumes of CTL or treatment groups were not different (B). Compared to CTL, the neuronal density of MD, NIC and NIC+MD groups were significantly decreased by 16.5±4.6%, *p<0.05* for MD; 12.9±3.0%, p<0.05 for NIC; 22.3±2.5%, *p<0.01* for NIC+MD) (C), *p<0.05; **p<0.01.

In comparison to the CA1 and CA3 regions of the hippocampus, the dentate gyrus develops relatively late, during the postnatal period, and retains its capability for ongoing neurogenesis through adulthood [11], [44]. This brain region would be undergoing active neurogenesis during the maternal deprivation periods, and would likely be affected by perturbations of development by stressors. However, NIC treatment caused no change in granule neuron number when compared to saline treated controls, whereas MD and NIC+MD groups were decreased by 14.1±1.4% and 15.9±2.0%, respectively (p<0.05, each), Fig. 6A. There was no distinction of DG volume between control and experimental groups (Fig. 6B). However, compared to control, the packing density of granule neurons was significantly lower for NIC, MD, and NIC+MD which, in comparison to control groups, respectively exhibited fewer neurons per mm^3^ by −12.9±3.0%, p<0.05 for NIC, −16.5±4.6%, p<0.05 for MD, and −22.3±2.5%, p<0.01 for NIC+MD (Fig. 6C, table 2). These data indicate that unlike in CA1 and CA3 regions where neuron numbers were significantly increased due to NIC or MD exposure, the DG responded to these developmental perturbations by a net reduction in neuron numbers, possibly as a result of depressed neurogenesis or increased apoptosis.

### Redistribution of Hippocampal Neurons

**Figure 7:**
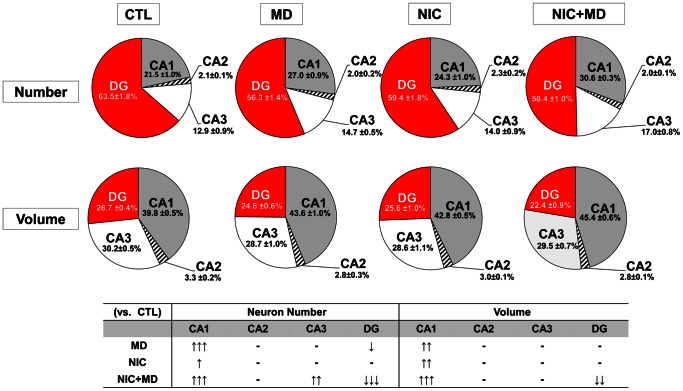
Fractional composition of CA1, CA2, CA3 and DG in P14 rat hippocampi after prenatal nicotine and/or maternal deprivation. The data for each hippocampal subregion is presented as a percentage of total neurons or neuronal volume of the hippocampus. The table summarizes data presented in pie graphs. Direction of arrows denotes relative increase (↑) or decrease (↓) in fractional composition of neuron number or volume compared to control animals. ↑ or ↓, p<0.05; ↑↑ or ↓↓, p<0.01; ↑↑↑ or ↓↓↓, p<0.001.

When all the neurons from CA1, CA2, CA3 and DG of the hippocampal formation were analyzed relative to one another, a prominent finding was that CA1 neurons made up 21.5±1.0% of total neurons in CTL, 27.0±0.9% in MD, 24.3±1.0% in NIC and 30.6±0.3% in NIC+MD. Theses changes in fractional distribution of hippocampal subregions were significant for MD (p<0.001), NIC (p<0.05) and NIC +MD (p<0.001) compared to control. Moreover, MD vs. NIC (p<0.05), MD vs. NIC+MD (p<0.01) and NIC vs. NIC+MD (p<0.001) were significant relative to each other. CA2 neurons remained unaffected at approximately 2% of total for the three treatment paradigms. CA3 neurons made up 12.9±0.9% of neurons in control hippocampi, and neither MD nor NIC significantly changed this percentage (p>0.05 for both). By contrast, the percentage of CA3 in the hippocampus was increased to 17.0±0.8% of total in MD +NIC brains (p<0.01). In controls, the DG makes up 63.5±1.8% of total neurons in the hippocampal formation. This percentage was decreased to 56.3±1.4% in MD (p<0.05), not affected in NIC 59.4±1.8% (p>0.05), and reduced to a mere 50.4±1.0% in NIC+MD treated animals (p<0.001). NIC+MD-induced reduction in DG was also significant compared to MD (p<0.05) or NIC (p<0.01) Fig. 7, top).

Analysis of the estimated volume associated with cell bodies of CA1 increased from 39.8±0.5% in controls to 43.6±1.0% in MD (p<0.01), 42.8±0.5% in NIC (p<0.05), or 45.4±0.6% in NIC+MD treated animals (p<0.001). CA1 volume for NIC+MD compared to NIC was also significantly expanded (p<0.05). The relative estimated volumes occupied by CA2 and CA3 pyramidal neurons was not affected by MD, NIC, or MD+NIC exposure. However, the percent estimated reference volume associated with cell bodies of DG granule neurons shrunk significantly in NIC+MD to 22.4±0.9% from the CTLs’ 26.7±0.4% (p<0.001), and in comparison to NIC 25.6±1.0% (p<0.05) and MD 24.8±0.6% (p<0.01). Neither NIC nor MD DG volumes were different from control or from each other (Fig. 7, bottom). Together, these data implicate that prenatal nicotine and maternal deprivation can induce drastic alterations in neural connectivity by perturbing the ratio of neurons in hippocampal subregions.

### Effects of Maternal Deprivation and Prenatal Nicotine are Prevalent in Rostral or Caudal Hippocampus

**Figure 8:**
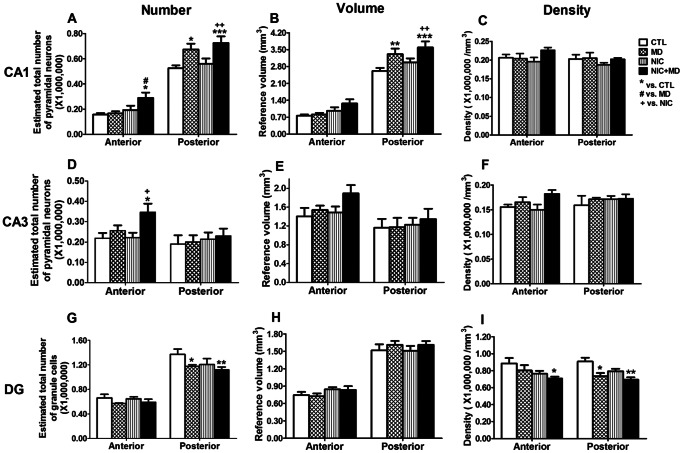
Preponderance of prenatal nicotine and maternal deprivation effects in rostral or caudal hippocampus of P14 rat. Synergistic interactions of prenatal nicotine and maternal deprivation on neuron number were present only in rostral CA1 and CA3 (A, D), which respectively showed increases of 84.7±26.8% and 58.8±19.0%, p<0.05 for both. Effects on CA1 pyramidal neuron number (A) and reference volume (B) specifically due to MD were visible only in caudal CA1 (*p<0.001* for NIC+MD vs. CTL and *p<0.05 or 0.01* for MD vs. CTL), and not CA3 (D, E), with no effect on density (C, F). DG granule cell number and density were decreased in caudal hippocampus due to MD or MD+NIC (G, I). Density of DG cells from NIC+MD groups was reduced in an additive manner (I). */#/+, p≤0.05; **/++, p≤0.01; ***p≤0.001.

The hippocampus is known to have regionally specific functions. For example, the most anterior/rostral regions are reported to respond to place cues during learning and memory for cognitive functions whereas the posterior/caudal-most regions are more readily associated with emotional and stress responses; reviewed in [45]. To determine the regional effects of MD and NIC, rostral and caudal hippocampus was analyzed. Using Paxinos’ atlas [39], neurons from hippocampal sections at or anterior to the Bregma −4.2 adult brain equivalent were designated rostral, whereas those at or posterior to the Bregma −4.36 adult brain equivalent were designated caudal. For rostral CA1 (Fig. 8A left), MD groups had 1.7±0.2×10^5^ neurons and NIC had 1.9±0.3×10^5^, and these were not statistically significant from each other or controls, which had 1.6±0.1×10^5^ neurons (p>0.05 in all cases). However, there was a significant, synergistically elevated number of pyramidal neurons when treatments were combined in NIC+MD (2.9±0.4×10^5^ neurons), an increase of 84.7±26.8% over control (p<0.05) and 74.2±25.2% over MD alone (p<0.05). Although NIC +MD neurons were greater than NIC by 50.2±21.8%, this difference did not reach significance, p>0.5, n.s. Analysis of caudal/ventral CA1 revealed that MD caused a significant increase of 28.2±8.7%, p<0.05 from control, whereas NIC had no effect on these neurons 6.7±7.7%, p>0.05. The combined NIC+MD treatment resulted in an increased number of neurons, 38.9±9.8%, p<0.001, relative to CTL, but this rise in neuron number was statistically different from NIC (p<0.01) but not MD (p>0.05).

The reference volumes were not significant from each other in rostral regions of the hippocampus, p>0.05 for all groups (Fig. 8B, left), whereas in caudal hippocampus, MD and MD+NIC caused respective 26.4±8.8%, p<0.01 and 37.1±9.5%, p<0.001 expansions. NIC, on the other hand, had no effect on volume, p>0.05 (Fig. 8B, right). Neuron density calculations showed no effect of treatment associated with either rostral or caudal CA1, p>0.05 for all groups (Fig. 8C). These provocative findings indicate that the MD-specific effect was restricted to the ventral, caudal hippocampus. By contrast, synergistic actions of prenatal nicotine combined with maternal deprivation primarily occurred on dorsal, rostral CA1 neurons (Fig. 8A–C).

Using the same parameters, we analyzed CA3 neurons for rostral or caudal preponderance of NIC and/or MD effects. The number of neurons in rostral CA3 of CTL, NIC, and MD groups were similar to each other, p>0.05. The only treatment with an effect was the combined NIC+MD treatment, which showed a synergistic augmentation of neurons over CTL; 58.9±19.0% (p<0.05). These neurons were 56.4±18.7% greater than NIC (p<0.05), and 35.7±16.2% (p>0.05) greater than MD (Fig. 8D). There was no effect of any treatment on caudal/ventral CA3 pyramidal neuron numbers (Fig. 8D). The reference volume of the rostral hippocampus were not statistically significant, although slightly increased for MD+NIC, p>0.05 (Fig. 8E). Nonetheless, this resulted in a zero net change in neuronal density for both rostral and caudal CA3 (Fig. 8F). These findings for CA3 neurons validate and strengthen the CA1 data, which support the idea that synergistic augmentation of hippocampal pyramidal neurons by NIC+MD treatment is strictly attributable to the rostral portions of the hippocampus.

Next, we evaluated whether the reduction of granule neurons in the DG (Fig. 6) is associated with either the rostral or caudal hippocampus. For MD, there was a statistically significant decline of granule neuron number in caudal, 14.3±2.1% (p<0.05), but not rostral DG. NIC had no statistical effect on either region. The combined NIC +MD treatment caused a significant reduction in caudal DG neurons, −18.4±3.4% (p<0.01), but not in rostral, compared to control (Fig. 8G). MD and MD+NIC were not statistically different from each other or from NIC (p>0.05 for each). The effect of NIC+MD on total DG neuron number was not additive or synergistic, but rather, closely mirrored the MD-only profile (Fig. 8G). The reference volume occupied by granule cell bodies was similar in controls and experimental groups at both the rostral and caudal regions, p>0.05 in all cases (Fig. 8H). A significantly reduced cell density was detected in the caudal DG for MD, −19.2±4.3% (p<0.05), and NIC+MD, −23.5±3.0% (p<0.05) compared to control but not compared to NIC. Neuron density in caudal hippocampus of NIC-treated animals was reduced by −12.7±3.1% compared to control, however there was no statistical significance compared to control or other groups (p>0.05) (Fig. 8I). Unexpectedly, NIC+MD but not MD- or NIC- treated rostral hippocampus had significantly decreased neuron densities relative to control, −20.2±2.6% (p<0.05). MD and NIC neuron density decreased by 9.4±7.1% (p>0.05) and 13.1±3.8% (p>0.05), respectively, but these were not statistically significant. The calculated drop in neurons from NIC+MD treatment was nearly additive in comparison to NIC or MD alone (Fig. 8I).

### Apoptosis and Neurogenesis in the DG

**Figure 9:**
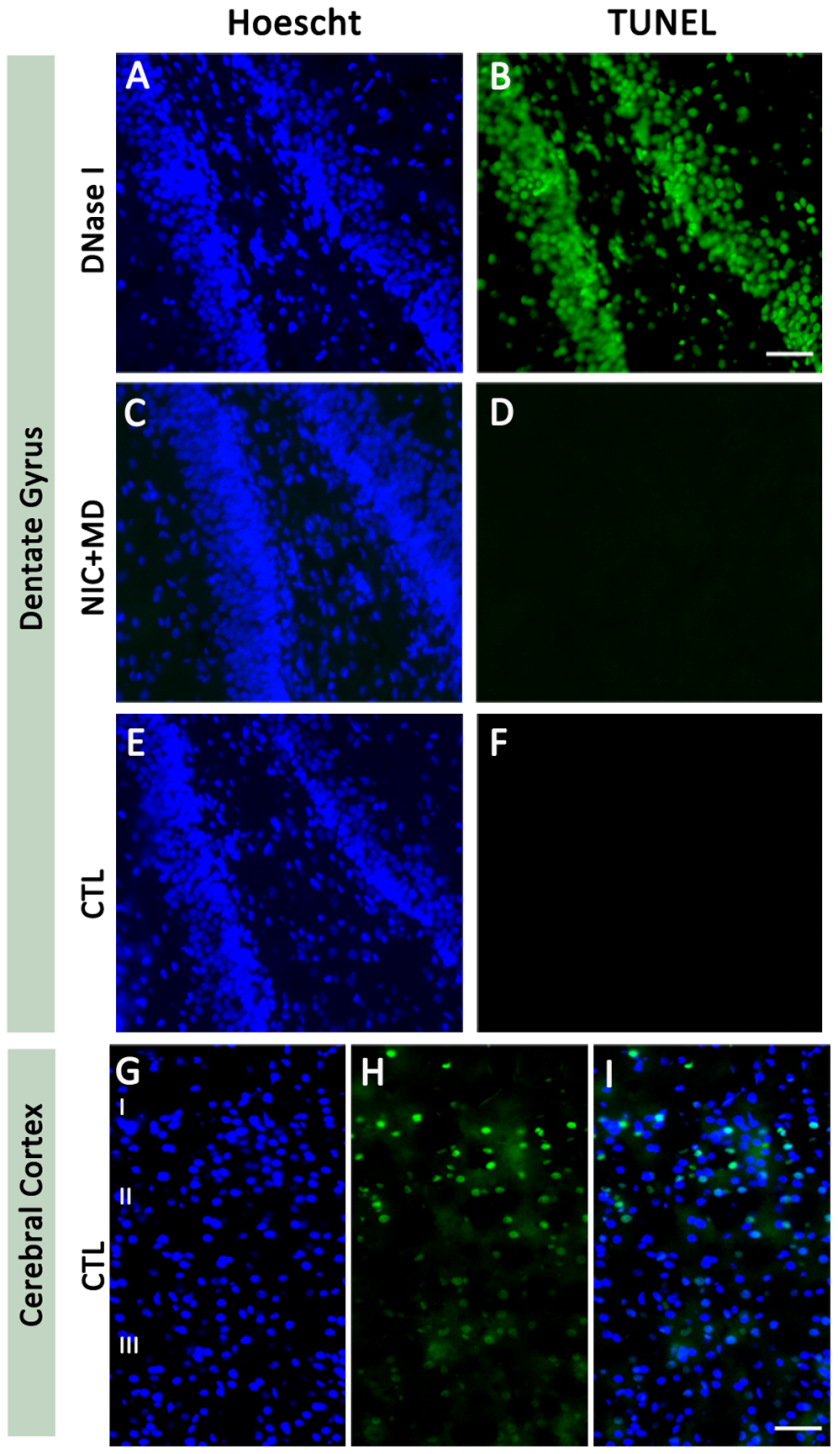
Detection of apoptotic cells in the dentate gyrus. Representative examples of Hoescht 33342-counterstained (A, C, E, G, I (blue)) TUNEL label (B, D, F, H, I (green)) in the dentate gyrus (A–F) or cerebral cortex (G–I) of P14 control (A, B, E–I) and MD+NIC (C, D) treated animals. Sections were treated with DNase 1 to generate DNA strand breaks, as a positive control (B). There was no TUNEL positive reaction in either NIC+MD (D) or in CTL (F) P15 DG. More superficial cortical layers had numerous cells that were positive for TUNEL as part of normal development (H, I). G–I served as a same section control for DG regions shown in E, F. Arrows point to endogenous TUNEL+ cells that have little to no reactivity for the Hoescht nuclear stain. Bars = 50 µm for all. I, II, III represent layers I, II, III of the cerebral cortex.

To examine if the observed reduction in neuron number and density noted in the dentate gyrus after NIC+MD treatment is due to actively apoptosing cells, 10 µm coronal sections of hippocampus were analyzed for DNA fragmentation using a terminal deoxyribonucleotidyl transferase (TDT)-mediated dUTP-digoxigenin nick end labeling (TUNEL) *in situ* assay. DNA strand breaks were introduced into CTL sections by exposure to DNAse I for ten minutes, and this served as a positive control for these studies. DNAse I treatment yielded positive staining for TUNEL in all cells detected with a Hoescht nuclear counterstain (Fig. 9A and 9B). There were no apoptotic cells in NIC+MD (Fig. 9C and 9D) treated hippocampi, and consistent with published findings [46], [47], in situ apoptosis was not detected for control hippocampus at P14 (Fig. 9E and 9F) (only DG is shown). By contrast, the cells in layer 2/3 of the cerebral cortex were positive for TUNEL-stained nuclei (Fig. 9G–9I). These data indicate that the NIC+MD treatment did not induce ongoing apoptosis in the hippocampus at the P14 time point analyzed, and were qualitatively no different from control.

**Figure 10:**
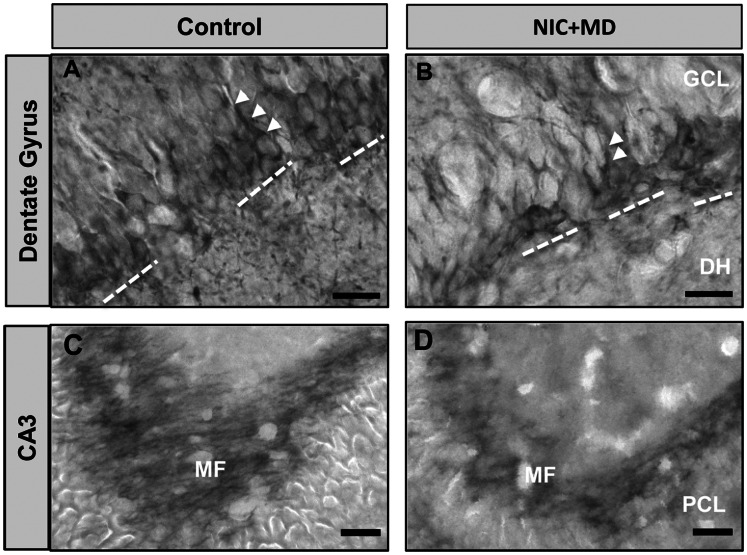
Doublecortin immunoreactivity in the developing dentate gyrus. Representative examples of DCX positive neuroblasts in control (A, C) and MD+NIC-exposed (B, D) P14 hippocampus. Differentiating DCX-positive cell bodies of DG (A, B) and mossy fiber projections to CA3 (C, D) are more abundant in control compared to MD+NIC. Arrowheads represent a DCX+ cell body and proximal dendrite in the plane of focus. Dashed bars are cohorts of DCX+ neurons in the subventricular zone. MF, mossy fiber; GCL, granule cell layer; DH, dentate hilus; PCL, pyramidal cell layer of CA3. Bar = 20 µm for A and B, and 50 µm for C and D.

To determine if the reduction in neurons of the DG induced by NIC+MD treatment was due to decreased neurogenesis, we evaluated DCX immunoreactivity in CTL and NIC+MD treated groups (fig. 10). Neurodifferentiation in the DG was ongoing and robust at the P14 age, and thus the DCX antibody labeled many cohorts of developing neurons (Fig. 10A) as well as their abundant mossy fiber projections to proximal dendrites of CA3 (Fig. 10C). Many neurons were immunoreactive to DCX in the developing DG of MD+NIC, but were qualitatively assessed to be fewer compared to the control neurons labeled (Fig. 10B), and their mossy fiber bundles projecting to CA3 were diminished (Fig. 10D). Therefore, it is plausible that the reduction in neuron number in DG could be due to reduced neurogenesis.

## Discussion

This is the first report investigating the combined effect of prenatal nicotine exposure and postnatal maternal deprivation stress on the brain. A unique aspect of this study is the analysis of these early life experiences at P14, a period of continued active development in the rat brain.

We report a synergistically increased number of pyramidal neurons in CA regions of P14 rat hippocampus, and concomitantly, a reduction of granule neuron number in the DG. Our data show that the synergistic effect of MD and NIC in CA1 and CA3 was attributable to changes in rostral/dorsal regions of hippocampus. In the DG, the combination of MD plus NIC additively decreased neuron density in the rostral/dorsal DG. Furthermore, neuron number and density in the caudal/ventral hippocampus in NIC+MD animals primarily reflected the effects of MD stress. Further, we show that the reduction in neurons observed in the DG of MD+NIC is correlated with reduced neurogenesis in that region.

### Laterality of the Hippocampus

Laterality of many brain functions is well established, but is not always considered. Studies of patients with mistreatment-induced PTSD and General Anxiety Disorder revealed a pronounced right>left asymmetry in the superior temporal gyrus gray and white matter volume, respectively [48], [49]. Similarly, structural and molecular laterality in the size of synapses, in the expression of NMDA and AMPA receptor subunits [7], [40], [41] as well as in the amplitude of EPSPs have been described for CA1 and CA3 of the hippocampus [7], [41]. In our studies, there was an initial concern that the treatment paradigm may have preferential effects on one side of the hippocampus versus the other. The data presented do not register a significant difference between the left and right side of the hippocampus in controls or with treatment although there is a trend toward fewer neurons in the right hippocampus compared to the left. Caution is still warranted when choosing experimental parameters for various studies. More directed experiments are needed to validate if the right hippocampus is more susceptible to insult than a more resistant left hippocampus – especially during development.

### Effect of Prenatal Nicotine in Hippocampal Development

We and others have shown that after gestationally restricted exposure to nicotine, the hippocampus continued to display long-lasting aberrant expression of synaptic modulators, inclusive of glutamate receptor subunits, during development, and these effects lasted into adulthood [14], [15], [50]. Likewise, maternal deprivation stress early in development has long lasting effects on genes affecting synaptic plasticity in adult brain [51], reportedly decreasing NR2a and NR2b receptor subunits [21], PSD95, and BDNF expression and function in adulthood [21], [24], [52].

The only other investigation of hippocampal cell numbers after prenatal nicotine exposure in the literature which uses unbiased stereological analysis reported no effect of prenatal nicotine on diverse neuronal populations of the adult hippocampus [30]. Our P14 study is in agreement in that there was no significant change in CA or DG neurons due to nicotine. However, this would not preclude abnormalities in behavior, neuronal function and molecular relationships at the synapse due to other NIC-induced perturbances in neuronal development [16], [50].

### Effect of Maternal Deprivation on Hippocampal Development

The weight of animals treated with MD or MD+NIC were suppressed compared to their NIC or CTL counterpoints. The time points when these weight differences were first observed coincide with the end of the stress hyporesponsive period during which there are low circulating levels of stress hormones and presumable non-responsiveness to external stressors [53], [54]. However, MD during the hyporesponsive period was documented to cause alterations in adult brain function [20], [55], [56], [57], [58]. Maternal deprivation stress alone induced a significant, rise in CA1 pyramidal neurons (Fig. 4A) and a decline of DG granule neurons (Fig. 6A), indicating distinct, contrasting effects of MD on these cell populations. Since CA1 neurons are already born at P2 when the separation began, it is possible that the MD experience delays progression of development, causing retention of neurons in an immature state; whereas, the postnatally born DG granule neurons can be more susceptible to effects on neurogenesis, accounting for the decreased number of neurons at P14 and the decreased reactivity to DCX. The mechanism of how MD affects neuron number is not yet known, but we cannot rule out the possibility that increases of CA1 pyramidal neurons could still be due to compensatory neurogenesis in response to MD, similar to CA1 undergoing neurogenesis following ischemic [59] or neonatal hypoxic injury [60]. Moreover, MD assessed in adults reportedly causes no long-term changes in CA1 or CA2/3 neuron number [29]. Thus, we may have captured a critical period of development when there is a massive upsurge of CA1 pyramidal neurons due to MD exposure, prior to stabilization of synapses.

A salient feature of the dentate gyrus is its ability to undergo neurogenesis not only during development, but also throughout adulthood. The DG is proposed to function in establishing and consolidating new memories, and can alter its neurogenesis functions dependent on activity, experience and mood. The MD-induced reduction in DG granule neurons in this P14 study (Fig. 6A) is fairly consistent with another stereology report of 20% reduction in mouse adult DG after a single 24hour MD [29] or an approximate 40% reduction in BrdU labeling in adult brain after chronic MD from P2–P21 [25]. We postulate that the reduction of granule neurons at P14 may be due to both impairment of DG neurogenesis and enhancement of apoptotic events because in adults exposed to MD, BrdU labeling was reduced [25], [61]. Importantly, our findings with DG also indicate that there may be a looser packing density with fewer neurons per area than controls. These findings suggest that DG perforant path connectivity or mossy fiber projections to CA3 could be re-structured, and induce permanent changes in neuroarchitecture.

### Combined Effect of Prenatal Nicotine and Maternal Deprivation

The findings of this study show that during development maternal deprivation stress can alter the ontogeny of CA1 and DG neurons, but not CA3. Our surprising finding was that in maternally deprived animals that had previously been exposed to gestational nicotine, a profoundly enhanced effect on pyramidal neurons was evident in CA1. Even more unexpected was that this combined treatment would result in such a robust increase in CA3, an area seemingly unaffected by neither MD nor NIC alone (Fig. 5). One possible explanation for the combined synergistic effects is that the neurons are already primed by the NIC exposure, and a second insult in the form of MD during susceptible periods of development exacerbates otherwise subtle effects on neuron number.

There is another report where animals exposed to MD for a single 24 hour period at P9 were subsequently exposed to cannabinoid agonists during adolescence, but data in this study show that when combined there was, for the most part, a reversal of any effect that either cannabinoid or MD alone might have on BDNF or CB1 receptor expression [62]. Thus, the timing of the drug exposure, as well as the type of drug of abuse is important in understanding how MD might enhance or repress brain development.

Another interesting finding in our model is the strong association with the rostral hippocampus, of synergistic (CA1, CA3) or additive (DG) consequences of developmental nicotine and stress exposure on neuron number (Fig. 8). The stress response enhanced subtle effects of nicotine specifically in the rostral hippocampus. By contrast, the caudal hippocampus seems to preferentially represent the MD component of the NIC+MD treatment. Many studies show functional segregation of the rostral/dorsal from the caudal/ventral hippocampus, where rostral regions are associated with cold cognitive, generic novelty learning, and caudal is associated with affective behaviors where behavioral relevance of stimuli is of importance [45], [63]. Thus, it is likely in our paradigm that MD and NIC together have a greater influence on cognitive learning via the rostral hippocampus whereas MD elicits affective or stressful influences via its actions on dorsal hippocampal neurons.

Moreover, we detected that a possible consequence of the combined treatment could be reduced neurogenesis in the dentate gyrus and reduced mossy fiber input to CA3. Indeed, upon analysis of DGs exposed to MD+NIC, which influences granule neuron numbers similarly to MD alone, there were reduced DCX positive neurons and DCX positive fibers (Fig. 10). A recent study confirms that MD can result in decreased DG neurogenesis at P15 [64]. MD exposure can also change the strength and abundance of neural circuits by causing cholinergic forebrain neurons to become vulnerable to subsequent damage [57], or as shown in this study and another [65], by reducing and perhaps weakening mossy fiber input to CA3. This could also result in altered connectivity of the Schaeffer collaterals to CA1. Although MD+NIC samples showed no detectable apoptosis (Figure 9), it is possible that an apoptotic cascade was induced earlier during the MD treatment or even during the prenatally restricted NIC treatment that was not caught at P14.

In conclusion, this initial investigation of the combined impact of gestational drug exposure with neonatal stress in the developing brain is provocative but raises several mechanistic questions. The fact that these treatments and exposures during the malleable prenatal and early postnatal periods could synergistically exacerbate perturbations of hippocampal development is an important consideration for further studies on drugs of abuse and stress as predictors of adolescent and adult cognition and affective disease. Additional studies are necessary to assess the functional consequences of these co-morbid conditions on behavior, and their impact on synaptic transmission, secretory mechanisms [66], and neural circuits during development and in adulthood.

## Supporting Information

Table S1 **Supplementary table for Bonferroni posthoc tests of animal weight.**
(DOCX)
