# Supplementary material for: Prenatal Nicotine and Maternal Deprivation Stress De-Regulate the Development of CA1, CA3, and Dentate Gyrus Neurons in Hippocampus of Infant Rats
Source: PLoS One. 2013 Jun 13;8(6):e65517. doi: 10.1371/journal.pone.0065517 (PMC3681797; doi:10.1371/journal.pone.0065517)
Supplement: Table S1 — Supplementary table for Bonferroni posthoc tests of animal weight. (DOCX) [file pone.0065517.s001.docx]

| Supplementary table for Bonferroni posthoc tests of animal weight | | | | | | | | | | | | | | | | | | | | | | |
| --- | --- | --- | --- | --- | --- | --- | --- | --- | --- | --- | --- | --- | --- | --- | --- | --- | --- | --- | --- | --- | --- | --- |
| Male(P2-P24) | |  |  |  |  |  |  | Female (P2-P24) | | | | | | |  | Female (P2-P24, PP-NIC) | | | | | | |
| Postnatal day | CTL vs MD | CTL vs NIC | CTL vs NIC+MD | MD vs NIC | MD vs NIC+MD | NIC vs NIC+MD |  | day | CTL vs MD | CTL vs NIC | CTL vs NIC+MD | MD vs NIC | MD vs NIC+MD | NIC vs NIC+MD |  | postnatal day | CTL vs MD | CTL vs NIC | CTL vs NIC-MD | MD vs NIC | MD vs NIC-MD | NIC vs NIC-MD |
|  |  |  |  |  |  |  |  |  |  |  |  |  |  |  |  | 1 | ns | ns | ns | ns | ns | ns |
| 2 | ns | ns | ns | ns | ns | ns |  | 2 | ns | ns | ns | ns | ns | ns |  | 2 | ns | ns | ns | ns | ns | ns |
| 4 | ns | ns | ns | ns | ns | ns |  | 4 | ns | ns | ns | ns | ns | ns |  | 4 | ns | ns | ns | ns | ns | ns |
| 6 | ns | ns | ns | ns | ns | ns |  | 6 | ns | ns | ns | ns | ns | ns |  | 6 | ns | ns | ns | ns | ns | ns |
| 8 | ns | ns | ns | ns | ns | *** |  | 8 | ns | ns | ns | ns | ns | ns |  | 8 | ns | ns | ns | ns | ns | ns |
| 10 | ns | ns | ns | ns | ns | *** |  | 10 | ns | ns | ns | ns | ns | * |  | 10 | ns | ns | ns | ns | ns | ns |
| 12 | ns | ns | ** | * | ns | *** |  | 12 | ns | ns | *** | * | ns | *** |  | 12 | ns | ns | ns | ** | ns | ns |
| 14 | ns | ns | *** | ** | ** | *** |  | 14 | ** | ns | *** | *** | ns | *** |  | 14 | ns | ns | ns | *** | ns | * |
| 16 | *** | ns | *** | *** | * | *** |  | 16 | *** | * | *** | *** | ns | *** |  | 16 | * | ns | ns | *** | ns | * |
| 18 | *** | ns | *** | *** | * | *** |  | 18 | *** | *** | *** | *** | ns | *** |  | 18 | * | ns | ns | ** | ns | * |
| 20 | *** | ** | *** | *** | ns | *** |  | 20 | *** | *** | *** | *** | ns | *** |  | 20 | *** | ns | *** | *** | ns | *** |
|  |  |  |  |  |  |  |  |  |  |  |  |  |  |  |  | 22 | *** | ns | ** | *** | ns | ** |
| 24 | ns | *** | *** | *** | *** | *** |  | 24 | ns | *** | ns | *** | ns | *** |  | 24 | ** | ns | * | *** | ns | * |
|  |  |  |  |  |  |  |  |  |  |  |  |  |  |  |  |  |  |  |  |  |  |  |
| Male (P24-P68) | |  |  |  |  |  |  | Female (P24-P68) | | | | | | |  |  |  |  |  |  |  |  |
| postnatal day | CTL vs MD | CTL vs NIC | CTL vs NIC+MD | MD vs NIC | MD vs NIC+MD | NIC vs NIC+MD |  | postnatal day | CTL vs MD | CTL vs NIC | CTL vs NIC+MD | MD vs NIC | MD vs NIC+MD | NIC vs NIC+MD |  |  |  |  |  |  |  |  |
| 24 | ns | ns | ns | ns | ns | ns |  | 24 | ns | ns | ns | ns | ns | ns |  |  |  |  |  |  |  |  |
| 28 | ns | ns | ns | ns | ns | ns |  | 28 | ns | ns | ns | ns | ns | ns |  |  |  |  |  |  |  |  |
| 32 | ns | ns | ns | ns | ns | ns |  | 32 | ns | ns | ns | ns | ns | ns |  |  |  |  |  |  |  |  |
| 36 | ns | ns | ns | ns | ns | ns |  | 36 | ns | ns | ns | ns | ns | ns |  |  |  |  |  |  |  |  |
| 40 | ns | ns | ns | ns | ns | ns |  | 40 | ns | ns | ns | ns | ns | ns |  |  |  |  |  |  |  |  |
| 44 | ns | ns | ns | ns | ns | ns |  | 44 | ns | ns | ns | ns | ns | ns |  |  |  |  |  |  |  |  |
| 48 | ns | ns | ns | ns | ns | ns |  | 48 | ns | ns | ns | ns | ns | ns |  |  |  |  |  |  |  |  |
| 52 | ns | ns | ns | ns | ns | ** |  | 52 | ns | ns | ns | ns | ns | ns |  |  |  |  |  |  |  |  |
| 56 | ns | ns | ns | ns | ns | *** |  | 56 | ns | ns | ns | ns | ns | ns |  |  |  |  |  |  |  |  |
| 60 | ns | ns | ns | ns | ns | *** |  | 60 | ns | ns | ns | ns | ns | ns |  |  |  |  |  |  |  |  |
| 64 | ns | ns | ns | * | ns | *** |  | 64 | ns | * | ns | ns | ns | ns |  |  |  |  |  |  |  |  |
| 68 | ns | * | ns | ** | ns | *** |  | 68 | ns | ns | ns | ns | ns | ns |  |  |  |  |  |  |  |  |

CTL, control; MD, maternal deprivation; NIC, prenatal nicotine only; MD+NIC prenatal nicotine and maternal deprivation; PP-NIC, the NIC treatment consists of prenatal nicotine + postnatal nicotine until P21. ns, p>0.05; *, p<0.05; **, p<0.01; ***, p<0.001
